# Left Atrial Appendage Closure for Stroke Prevention in Patients with Atrial Fibrillation and Hereditary Hemorrhagic Telangiectasia

**DOI:** 10.1155/2012/646505

**Published:** 2012-09-03

**Authors:** Sebastiaan Velthuis, Martin J. Swaans, Johannes J. Mager, Benno J. W. M. Rensing, Lucas V. A. Boersma, Martijn C. Post

**Affiliations:** ^1^Department of Cardiology, St. Antonius Hospital, 3435 CM Nieuwegein, The Netherlands; ^2^Department of Pulmonology, St. Antonius Hospital, 3435 CM Nieuwegein, The Netherlands

## Abstract

Atrial fibrillation (AF) is the most common cardiac arrhythmia, affecting millions of individuals worldwide, and a major risk factor for disabling cerebral embolic stroke. Hereditary hemorrhagic telangiectasia (HHT) is an autosomal dominant inherited disorder, characterized by vascular abnormalities with high-bleeding tendency and therefore intolerance for oral anticoagulation. We report a case of percutaneous closure of the left atrial appendage, which might be a good alternative strategy instead of chronic oral anticoagulation to protect patients with high-risk AF and HHT from cerebral embolic strokes.

## 1. Introduction

Atrial fibrillation (AF) is the most common cardiac arrhythmia and a major risk factor for cerebral embolic stroke. Although oral anticoagulation (OAC) is highly effective in stroke prevention, a substantial number of patients is unable to sustain chronic OAC because of high-bleeding risks. Among these are patients with hereditary hemorrhagic telangiectasia (HHT), who frequently encounter inconvenient epistaxis, gastrointestinal bleedings, or life-threatening bleedings from cerebral or pulmonary arteriovenous malformations. Since most thromboembolic complications in patients with AF arise from the left atrial appendage (LAA), a percutaneous transcatheter LAA closure device was recently developed. 

This is the first paper about the feasibility of percutaneous LAA closure in patients with high-risk AF and HHT, which can be a promising strategy to protect these patients against embolic stroke, while avoiding the need for long-term OAC.

## 2. Case Presentation

**Figure 1:**
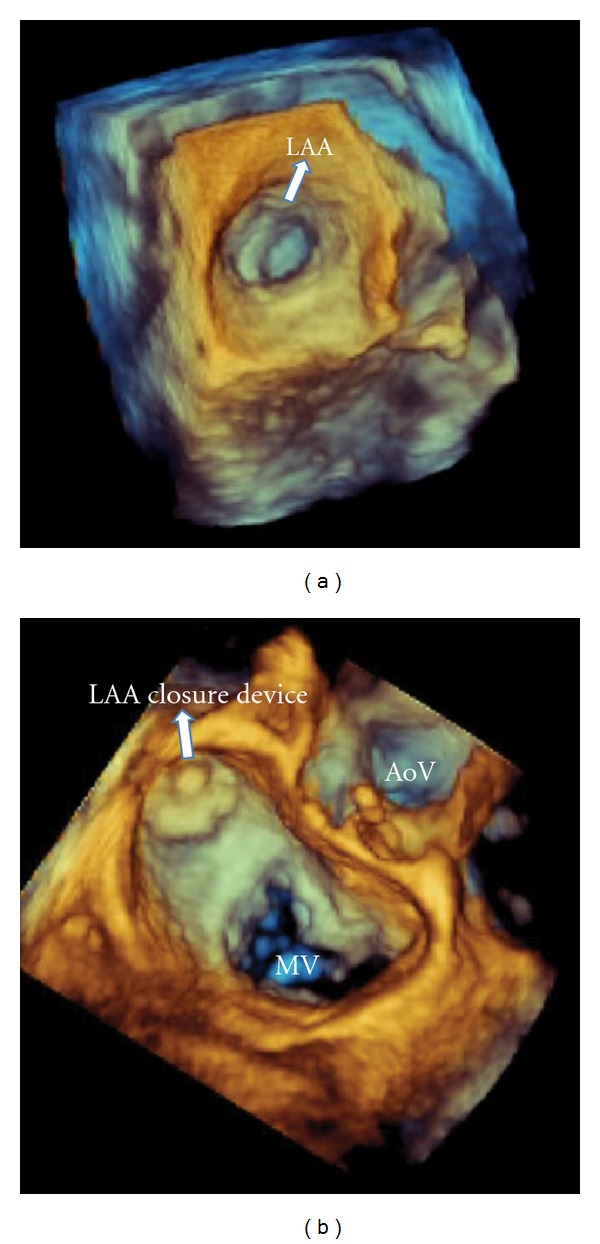
Real-time three-dimensional transoesophageal echocardiographic imaging of the left atrial appendage as seen from the left atrial perspective. (a) Preprocedural view of LAA. (b) LAA closure device deployed. (LAA: left atrial appendage; AoV; aortic valve; MV; mitral valve).

**Figure 2:**
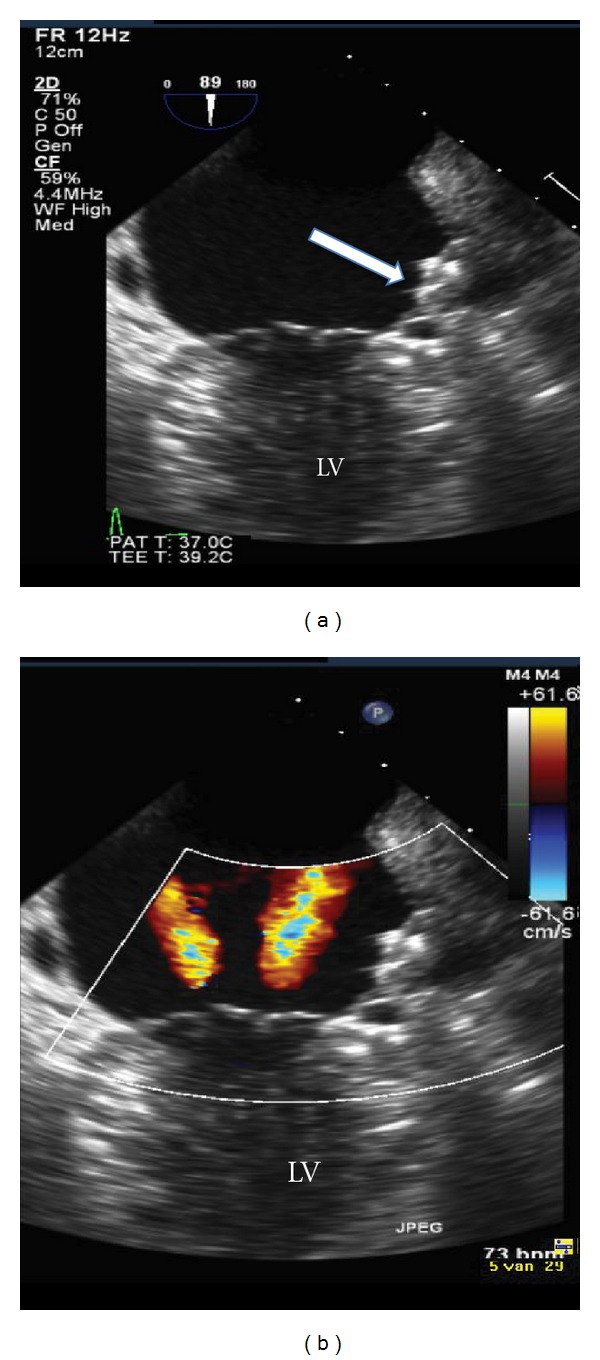
Two-dimensional transoesophageal echocardiographic imaging of the left atrium at 45-days followup. (a) Successful occlusion of the LAA without thrombus formation on the atrial surface of the device despite the limited use of anticoagulation therapy (aspirin only). (b) No residual flow around the device. LV: left ventricle.

We present a 79-year-old man who had been previously diagnosed with HHT, based on the clinical Curaçao criteria [1]; his family history revealed numerous first-degree relatives with HHT, physical examination demonstrated several telangiectasia on hands, face, lips, and ears, and he suffered from recurrent epistaxis. DNA analysis confirmed the clinical diagnosis with an ALK-1 gene mutation on chromosome 12. Visceral AVMs in the brain, lungs, liver, or gastro-intestinal tract were excluded with magnetic resonance imaging, echocardiography, computed-tomography, and endoscopy. In addition to HHT, the patient was known with arterial hypertension, a recent cerebral ischemic stroke, and permanent AF. His CHADS_2_-score of four indicated a high risk of recurrent stroke (8.5%/year) [2]. Since visceral sources of life-threatening hemorrhages were ruled out, a trial of OAC (warfarin) was started. This resulted in extensive epistaxis, requiring repeated blood transfusions despite treatment with iron supplements and nasal cauterizations. Because of this clinical dilemma, the patient was referred to our HHT-specialized cardiology department and was accepted for percutaneous LAA closure to protect against recurrent stroke, while avoiding the need for long-term OAC. A 27 mm Watchman LAA Occlusion Device (Atritech Inc., Plymouth, Minnesota) was implanted in the LAA using biplane fluoroscopy and 3D-transoesophageal echocardiography (TEE) guidance, according to the recent literature (Figure 1) [3]. Because of the patient's high-bleeding risk, postprocedural anticoagulation was limited to aspirin for at least six weeks to allow for device endothelialization and to prevent thrombotic complications. Control TEE at 45 days demonstrated successful closure of the LAA without thrombus formation on the atrial surface of the device (Figure 2), and aspirin was discontinued. During one-year followup, no thromboembolic complications or severe HHT-related bleedings occurred.

## 3. Discussion

This is the first paper about the feasibility of a percutaneous LAA closure device in patients with high-risk AF and HHT. Percutaneous closure of the LAA might be a new strategy to protect this population against cerebral embolic stroke without the need for chronic OAC and subsequent high-bleeding risks. 

HHT-related gene mutations result in abnormal angiogenesis and fragile vessels with increased bleeding tendency [4]. Recurrent epistaxis is the most common symptom of HHT, affecting 78–96% of patients [5]. Epistaxis often leads to iron-deficiency anemia and is an important factor reducing quality of life [6]. Gastro-intestinal (GI) telangiectasia is present in 80% of HHT patients, from which 30% will develop GI-bleedings and causes increased morbidity and mortality [7]. Approximately 23% of HHT patients have cerebral AVMs. The bleeding risk from cerebral AVMs in HHT is estimated around 0.5% per year [8], which may have devastating consequences. Pulmonary AVMs are present in up to 58% of HHT patients [9] and carry the risk of hemoptysis or hemothorax, which is caused by a ruptured endobronchial or subpleural AVM and reported in up to 8% [10].

Because of this inconvenient and sometimes life-threatening bleeding tendency, the decision to start OAC in patients with high-risk AF and HHT should be based on a careful individual risk assessment of both thromboembolic and hemorrhagic complications. 

Previous studies demonstrated that the LAA is the source of thrombi in more than 90% of patients with nonvalvular AF [11], which led to the development of the Watchman left atrial appendage occlusion device. This percutaneous transcatheter device excludes the LAA from the systemic circulation and protects against embolic stroke, without the need for long-term OAC. The Watchman device was investigated in the PROTECT-AF trial, which demonstrated that percutaneous closure of the LAA was noninferior to chronic OAC therapy [3]. After device implantation, all patients in the PROTECT-AF trial were treated with warfarin for 45 days, clopidogrel and aspirin for six months, and then aspirin lifelong. Therefore, the exact safety and efficacy of percutaneous LAA device closure in patients with high-bleeding risks and intolerance for short-term OAC remains unknown. At our institution, HHT patients must be eligible for at least 6 weeks of aspirin therapy, which is tested before device implantation using a nosebleed diary. A careful risk-benefit assessment is made prior to LAA device implantation for every individual with HHT, in order to arrange a tailor-made postprocedural anticoagulation treatment. We underscore that the optimum strategy to prevent device-related thromboembolic complications without raising the risk of bleeding in patients with contraindications for OAC has to be established in future studies. At present, percutaneous LAA occlusion may be an acceptable option in selected HHT patients with high-risk AF who are not candidates for long-term OAC.

## 4. Conclusion

 Percutaneous closure of the LAA might provide an alternative strategy to chronic anticoagulation therapy for stroke prevention in patients with high-risk AF and HHT. 

## References

[1] Shovlin CL, Guttmacher AE, Buscarini E (2000). Diagnostic criteria for hereditary hemorrhagic telangiectasia (Rendu-Osler-Weber syndrome). *American Journal of Medical Genetics*.

[2] Camm AJ, Kirchhof P, Lip GY (2010). Guidelines for the management of atrial fibrillation: the Task Force for the Management of Atrial Fibrillation of the European Society of Cardiology (ESC). *European Heart Journal*.

[3] Holmes DR, Reddy VY, Turi ZG (2009). Percutaneous closure of the left atrial appendage versus warfarin therapy for prevention of stroke in patients with atrial fibrillation: a randomised non-inferiority trial. *The Lancet*.

[4] Fernandez-L A, Sanz-Rodriguez F, Zarrabeitia R (2005). Blood outgrowth endothelial cells from Hereditary Haemorrhagic Telangiectasia patients reveal abnormalities compatible with vascular lesions. *Cardiovascular Research*.

[5] Plauchu H, de Chadarevian JP, Bideau A, Robert JM (1989). Age-related clinical profile of heredity hemorrhagic telangiectasia in an epidemiologically recruited population. *American Journal of Medical Genetics*.

[6] Pasculli G, Resta F, Guastamacchia E, di Gennaro L, Suppressa P, Sabbà C (2004). Health-related quality of life in a rare disease: hereditary hemorrhagic telangiectasia (HHT) or Rendu-Osler-Weber Disease. *Quality of Life Research*.

[7] Longacre AV, Gross CP, Gallitelli M, Henderson KJ, White RIJ, Proctor DD (2003). Diagnosis and management of gastrointestinal bleeding in patients with hereditary hemorrhagic telangiectasia. *American Journal of Gastroenterology*.

[8] Willemse RB, Mager JJ, Westermann CJJ, Overtoom TTC, Mauser H, Wolbers JG (2000). Bleeding risk of cerebrovascular malformations in hereditary hemorrhagic telangiectasia. *Journal of Neurosurgery*.

[9] van Gent MWF, Post MC, Snijder RJ, Westermann CJJ, Plokker HWM, Mager JJ (2010). Real prevalence of pulmonary right-to-left shunt according to genotype in patients with hereditary hemorrhagic telangiectasia: a transthoracic contrast echocardiography study. *Chest*.

[10] Ference BA, Shannon TM, White RIJ, Zawin M, Burdge CM (1994). Life-threatening pulmonary hemorrhage with pulmonary arteriovenous malformations and hereditary hemorrhagic telangiectasia. *Chest*.

[11] Blackshear JL, Odell JA (1996). Appendage obliteration to reduce stroke in cardiac surgical patients with atrial fibrillation. *Annals of Thoracic Surgery*.

